# Supplementary material for: Patient safety culture in resource-limited healthcare settings: A multicentre survey
Source: PLoS One. 2025 Jun 25;20(6):e0326320. doi: 10.1371/journal.pone.0326320 (PMC12193601; doi:10.1371/journal.pone.0326320)
Supplement: S1 Table — (DOCX) [file pone.0326320.s001.docx]

**S1 Table**: Proportional allocation of participants in each Hospital, Eastern Ethiopia.

| Hospital | Total healthcare professionals | Sample allocated |
| --- | --- | --- |
| A | 612 | 288 |
| B | 186 | 88 |
| C | 165 | 77 |
| D | 308 | 145 |
| E | 187 | 88 |
| **Total** | **1,458** | **686** |
