# Supplementary material for: Patient safety culture in resource-limited healthcare settings: A multicentre survey
Source: PLoS One. 2025 Jun 25;20(6):e0326320. doi: 10.1371/journal.pone.0326320 (PMC12193601; doi:10.1371/journal.pone.0326320)
Supplement: S3 Table — (DCOX) [file pone.0326320.s003.docx]

**S3 Table:** The mean score of patient safety culture dimensions across clinical units.

| Dimensions |  | | Clinical units | | | | | | | | |  |
| --- | --- | --- | --- | --- | --- | --- | --- | --- | --- | --- | --- | --- |
|  | Medical wards | Surgical wards | | Ob/Gyn wards | Paediatric wards | ICUs | PDs | EOPDs | Overall mean | Welch F(df1,df2) | *P-Value* | η² |
| Teamwork | 3.93 + 0.79 | 3.65 + 0.81 | | 3.88 + 0.80 | 3.87 + 0.63 | 3.56 + 0.82 | 3.81 + 0.68 | 3.98 + 0.66 | 3.79 + 0.77 | 4.13(6,220.89) | *<0.001* | 0.043[0.01 -0.07] |
| Staffing and work pace | 3.23 + 0.61 | 3.13 + 0.61 | | 3.19 + 0.60 | 3.02 + 0.60 | 3.03 + 0.50 | 3.26 + 0.49 | 3.13 + 0.57 | 3.13+ 0.57 | 2.26(6,217.13) | *0.039* | 0.021[0.00-0.039] |
| Organisational learning and continuous improvement | 3.54 + 0.92 | 2.91 + 1.19 | | 3.25 + 0.85 | 3.12+ 1.06 | 2.72 + 1.23 | 3.39 + 0.77 | 3.35 + 0.76 | 3.15 + 1.04 | 7.21(6,219.28) | *<0.001* | 0.080[0.035-0.116] |
| Response to Error | 3.06+ 0.59 | 2.86+ 0.59 | | 3.03+ 0.54 | 3.00+ 0.58 | 2.85 + 0.54 | 3.10 + 0.55 | 3.09 + 0.54 | 2.98 + 0.57 | 3.38(6,217.18) | *0.003* | 0.034[0.005-0.058] |
| Supervisor, manager or clinical leader support for patient safety | 3.27 + 0.83 | 3.07 + 0.88 | | 3.10 + 0.91 | 3.05 + 0.97 | 2.75 + 0.78 | 3.27 + 0.78 | 3.32 + 0.77 | 3.09 + 0.89 | 5.07(6,218.01) | *<0.001* | 0.053[0.016-0.084] |
| Communication about error | 3.25+1.21 | 3.23+ 1.17 | | 3.54 + 1.17 | 3.30 + 1.14 | 3.93 + 1.09 | 3.62 + 1.01 | 3.63 + 0.96 | 3.32+ 1.13 | 5.76(6,217.42) | *<0.001* | 0.052[0.016-0.082] |
| Communication openness | 3.22 + 1.01 | 3.06 + 1.07 | | 3.23+ 0.97 | 3.16+ 1.14 | 2.75 + 1.08 | 3.36 + 0.85 | 3.40 + 0.83 | 3.13 + 1.02 | 5.17(6,217.80) | *<0.001* | 0.051[0.015-0.081] |
| Reporting patient safety incident | 3.12 + 1.13 | 2.87 + 1.18 | | 3.15 + 1.14 | 2.94 + 1.18 | 2.69 + 1.01 | 3.05 + 1.09 | 3.11 + 0.97 | 2.97 + 1.10 | 2.74(6,215.75) | *0.014* | 0.026[0.001-0.047] |
| Hospital management support for patient safety | 3.10 + 0.97 | 2.84 + 0.96 | | 2.99 + 1.01 | 2.81 + 0.95 | 2.66 + 1.02 | 3.31 + 0.82 | 3.09 + 0.77 | 2.95 + 0.96 | 4.64(6,218.34) | *<0.001* | 0.045[0.011-0.073] |
| Hands off and information exchange | 3.08+ 0.82 | 2.68+ 0.74 | | 3.04+ 0.75 | 2.76+ 0.80 | 2.57+ 0.78 | 3.19+ 0.88 | 3.04+ 0.70 | 2.88+ 0.80 | 8.01(6,216.48) | *<0.001* | 0.080[0.036-0.117] |

***Note:*** *EOPD=Emergency and outpatient department, ICU=Intensive care unit, Ob/Gyn=Obstetrics and Genecology, PD= Pharmacy and diagnostic unit, SSPS= Supervisor, Manager, or Clinical Leader Support for Patient Safety*
