# Supplementary figures and images for: Patient safety culture in resource-limited healthcare settings: A multicentre survey
Source: PLoS One. 2025 Jun 25;20(6):e0326320. doi: 10.1371/journal.pone.0326320 (PMC12193601; doi:10.1371/journal.pone.0326320)

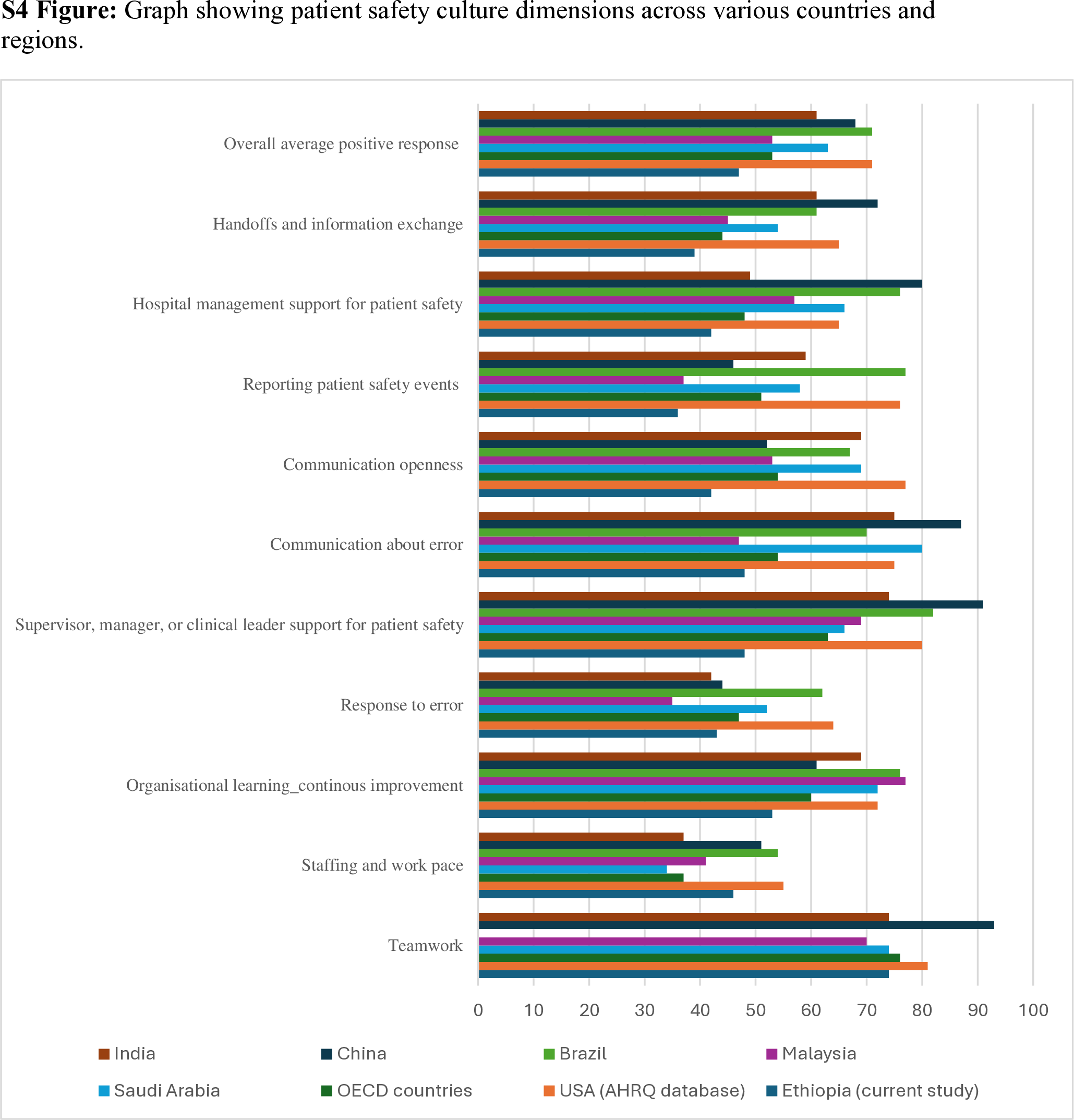

Supplement: S4 Fig — (TIF) [file pone.0326320.s004.tif]
