# Supplementary material for: Patient safety culture in resource-limited healthcare settings: A multicentre survey
Source: PLoS One. 2025 Jun 25;20(6):e0326320. doi: 10.1371/journal.pone.0326320 (PMC12193601; doi:10.1371/journal.pone.0326320)
Supplement: Attachment — Submitted filename: revivew respo for plos one.docx [file pone.0326320.s005.docx]

Thank you to look over my insight full expertise on this research work entitled “Patient safety culture in resource-limited settings: insights from healthcare professionals through a multi-center survey.” and I got this article is very interesting and thought full and identifying deferent Hedin factors that identified Patient safety culture in resource-limited settings.

The research work is very interesting and thought full for scholars and also leading factor for other researchers and also hospital administrators farther more for ministry of health

1. How do you measure Cronba ch’s α and what is the cut point of Cronba ch’s α?
2. What is your base line that you said that the patient safety is present or absent in the hospital? with reference
3. Why you were select the linear model for your research? Clearly enplane
4. Inline285 your finding “Compared with supervisors and other clinical staff, midwives were significantly less likely to rate patient safety positively,” why this is happened? Would you explain the tool you used (if you can tool).
5. Do you think that “lack of a patient safety incident reporting system,” is the main challenge is this how do you measure?
6. I do have question on your exclusion criteria do you think that the absent of study participant during study period exclusion criteria?

#. Do you think Is it possible two ethical clearance for one research work?

General comment for researchers I have read your finding and which is very good work but I recommended your study population was patients because the key stakeholder due to understand or answer your research title the actors were better to take patients
