# Supplementary material for: Patient safety culture in resource-limited healthcare settings: A multicentre survey
Source: PLoS One. 2025 Jun 25;20(6):e0326320. doi: 10.1371/journal.pone.0326320 (PMC12193601; doi:10.1371/journal.pone.0326320)
Supplement: Attachment — Submitted filename: Review.docx [file pone.0326320.s006.docx]

**Dear sir,**

The study examined ‘Patient safety culture in resource-limited settings: insights from healthcare professionals through a multi-centre survey’. This manuscript is well-written and sound, offering a significant understanding of patient safety culture in Ethiopian public hospitals. The study effectively identifies gaps in patient safety culture and provides actionable recommendations for improving it. However, several areas could be enhanced. Overall, with minor improvements, this manuscript has the potential to contribute significantly to the field of patient safety, particularly in resource-limited settings.

**Introduction**

The transition from HSoPSC 1.0 to 2.0 is mentioned, but the discussion on how HSoPSC 2.0 improves explicitly upon the previous version could be expanded. More concrete examples of its improvements, such as added dimensions or clarified questions, would enhance the reader’s understanding.

Again, while the introduction mentions Ethiopia’s under-researched state, a deeper exploration of the Ethiopian healthcare system’s specific challenges could enrich the introduction. For instance, referencing infrastructure deficits, regional disparities, and the socio-political climate in Ethiopia might provide more context for the gap in research.

**Methods and Materials**

1. The use of “consecutive sampling” is mentioned, but the rationale behind this choice could be better explained. For example, it could clarify why this technique minimizes selection bias and is suited for the context of Ethiopian public hospitals.
2. Was the tool pre-tested? While the HSoPSC 2.0 tool was adapted to local contexts, it would be helpful to discuss the specific modifications made to the tool (e.g., changes to the wording of questions or scale adaptation) to ensure its appropriateness for Ethiopian healthcare settings.

**Results**

1. The manuscript mentions “small to medium effect sizes (η²)” but does not provide specific values, which would help the reader interpret the magnitude of differences observed.
2. The manuscript should emphasize the practical significance of findings and statistical significance. For example, it could describe the implications of midwives’ lower ratings of patient safety culture beyond the statistical significance.
3. Why content analysis? Which questions demanded open-ended responses?
4. Which bivariate analysis was done? Correlation? Any results for that?

**Discussion**

1. While the study draws comparisons with other settings, a more profound synthesis of the results with existing theoretical frameworks on patient safety culture would strengthen the discussion. Connecting the results to safety culture models could add depth to the interpretation.
2. Some limitations, such as the poor internal consistency of specific subscales, are briefly mentioned. These should be more deeply integrated into the discussion, particularly in how they affect the interpretation of the findings and the robustness of the conclusions.

**Strengths and Limitations**

The internal consistency issues with several key subscales (e.g., Staffing and Work Pace) should be addressed more critically. A more precise explanation of how these limitations might have impacted the findings and their practical implications is needed.

**Conclusion and Implications**

While the conclusion outlines essential policy and practice recommendations, it could benefit from more emphasis on the long-term sustainability of these changes. For example, how might these recommendations be scaled or maintained over time, especially in resource-limited settings?

The call for future research could be more specific. For instance, it would be beneficial to suggest follow-up studies focusing on the long-term impact of the interventions recommended in this study.
