# Supplementary material for: Patient safety culture in resource-limited healthcare settings: A multicentre survey
Source: PLoS One. 2025 Jun 25;20(6):e0326320. doi: 10.1371/journal.pone.0326320 (PMC12193601; doi:10.1371/journal.pone.0326320)
Supplement: Attachment — Submitted filename: Response to Reviwers.docx [file pone.0326320.s007.docx]

**Point-by-point response to Editor and Reviewer’s Comments**

| **Editor and reviewers** | **Comments** | **Author responses** |
| --- | --- | --- |
| Editor’s comment | Please address the reviewers' comments, especially those related to benchmarking, methodological robustness and transparency and thematic integration. | Thank you for your valuable feedback, which has been instrumental in improving the quality of our manuscript. We have carefully and thoroughly addressed each of the reviewers' comments point-by-point and have incorporated all necessary revisions into the updated manuscript, which has been labelled as '**Manuscript'** in the submission system. |
| **Reviewer #1** | I would like to thank you for submitting this insightful paper on patient safety culture. This is an incredibly important topic, and your research provides valuable perspectives that can help advance the understanding and improvement of safety protocols in healthcare settings. The paper is well-written and highlights several crucial aspects of patient safety. I do have a few minor comments, specifically regarding the sample size and some potential gaps in the research that could be explored further. | Your feedback is highly appreciated and has been instrumental in refining our work. We have provided the responses point-by-point as follows; |
|  | **1. Sample size and sampling technique** Would you please clarify how the sample size was proportionally allocated to each hospital based on the total number of staff and how much was given to each hospital. | Thank you for this important question. The total sample size was proportionally allocated across the five participating hospitals based on the total number of healthcare professionals employed at each site using the formula: *Sample from hospital A=(Total number of healthcare professionals in Hospital A/total number of healthcare professionals in all hospitals)*Total sample size (686).* This proportional allocation ensured that larger hospitals contributed a correspondingly greater share of participants, thereby enhancing the representativeness of the sample. To improve the clarity and transparency on this regard we have included the total number of healthcare professionals at each hospital, and the proportionally allocated sample to each hospital as follows” Accordingly, from Hospital A, out of a total of 612 healthcare professionals, 288 were proportionally allocated. For Hospital B, 186 professionals were available and 88 were allocated; Hospital C had 165 professionals with 77 allocated; Hospital D had 308 with 145 allocated; and Hospital E had 187 with 88 allocated**.”(Line 155-160).** |
|  | **2. Conclusion** It is worth stating the gaps this study has covered and any other gaps that should be investigated in the future. | We sincerely appreciate your insightful feedback. We have refined the conclusion section and included the contributions of our study **(lines 632–642),** and the recommendations for future research es including the longitudinal studies to track a change in patient safety culture over time and their impact on patient outcome, in-depth qualitative studies exploring why patient safety culture is lower in intensive care units compared to other units?, psychometric evaluations of the Hospital Survey on Patient Safety Culture 2.0 (HSoPSC 2.0) tool, interventional studies to examine effectiveness of strategies to enhance patient safety culture in resource limited settings along with follow-up studies to assess their scalability and sustainability in such settings. The details of recommendations for future research are highlighted from **Line 620-627**. |
| **Reviewer #2** | The paper provides valuable insights into patient safety culture in resource-limited settings, particularly in Ethiopian public hospitals. The study is well-designed, with a correct methodology responding to the study objectives. However, there are some limitations such as: | Your feedback is highly appreciated and has been instrumental in refining our work. We also appreciate your recognition of the importance of addressing these limitations. |
|  | • The low Internal Consistency of Subscales: Several subscales in the HSoPSC 2.0 showed poor internal consistency (Cronbach’s α < 0.70), which may have affected the reliability of the results. This limitation is acknowledged by the authors but could impact the validity of the findings. | We thank the reviewer for highlighting this important issue. We agree that low internal consistency in several subscales—particularly *Staffing and Work Pace* (α = .19) and *Handoffs and Information Exchange* (α = .48)—poses a significant methodological limitation. In response, we have revised the limitations section to explicitly state how this measurement issue may have affected the results. Specifically, we now clarify that these low alpha values may have obscured true effects in both the ANOVA and ordinal logistic regression analyses, potentially resulting in non-significant findings where meaningful relationships may exist. This measurement error increases the risk of Type II errors and may have led to the underestimation of effect sizes. The revised discussion **(Lines 554-569)** reflects this concern in detail and recommends future scale refinement. |
|  | Language Barrier : Although English is the medium of instruction in Ethiopian hospitals, some participants may have faced challenges in understanding the questionnaire, potentially affecting their responses. | We agree that language has the potential to influence how questions may have been answered and had included this as a key limitation of our work. We appreciate the reviewer’s recognition of this important issue. |
| **Reviewer #3** | Thank you to look over my insight full expertise on this research work entitled “Patient safety culture in resource-limited settings: insights from healthcare professionals through a multi-center survey.” and I got this article is very interesting and thought full and identifying deferent hidden factors that identified Patient safety culture in resource-limited settings. The research work is very interesting and thought full for scholars and also leading factor for other researchers and also hospital administrators farther more for ministry of health | Thank you for your feedback. We highly appreciate and it has been instrumental in refining our work. |
|  | 1. How do you measure Cronbach’s α and what is the cut point of Cronbach’s α? | The Cronbach’s alpha to assess the reliability of patient safety culture dimensions were calculated by using the using IBM SPSS Statistics for Windows version 30.0 (IBM Corp., Armonk, NY, USA) as outlined in **Table 2.** Cronbach’s alpha is a coefficient that estimates the proportion of variance in a scale score that is attributable to the true score, rather than to measurement error. It is calculated based on the average inter-item correlations and the number of items in the scale. Interpretation, values of Cronbach’s alpha are generally evaluated as follows:  ≥ .90 = Excellent  .80–.89 = Good  .70–.79 = Acceptable  .60–.69 = Questionable  < .60 = Poor  While many researchers adopt a cut-off of ≥ .70 as acceptable reliability (Fornell & Larcker, 1981; Hair et al., 2019), available at: <https://doi.org/10.1177/002224378101800313> and <https://thuvienso.hoasen.edu.vn/handle/123456789/10308> respectively. The developers of the Hospital Survey on Patient Safety Culture (HSoPSC 2.0) consider a Cronbach’s alpha of ≥ .60 to be acceptable for their instrument, given the conceptual breadth and diversity of items within each dimension (AHRQ User Guide). Our evaluation followed this guidance, which is consistent with psychometric practices for broad constructs. |
|  | 2. What is your base line that you said that the patient safety is present or absent in the hospital? with reference. | Thank you for raising this important point. According to the HSoPSC 2.0 user’s guide there is no baseline to categorise patient safety culture is “present” or “absent” in the hospital. However, the patient safety rating can be categorised as developed (≥75% positive responses), poorly developed (50–75% positive responses), and underdeveloped (<50% positive responses). Available at: <https://doi.org/10.1186/s40886-017-0062-9> and <https://www.ahrq.gov/sops/surveys/hospital/index.html>. Therefore, we have followed the user’s guide recommendations to categorise the patient safety culture as outlined in the manuscript **Line 240-242.** |
|  | 3. Why you were select the linear model for your research? Clearly explain | We sincerely appreciate your thoughtful feedback. To clarify, we did **not** use a linear regression model, as our outcome variable—patient safety rating—was measured on an **ordinal Likert scale** ranging from “poor” to “excellent.” A linear model would have been inappropriate, as it assumes a continuous dependent variable with equal intervals between values. Instead, we employed **ordinal logistic regression (OLR)**, which is specifically suited for ordinal outcomes because it models the log-odds of being at or below a certain category while preserving the rank order of the responses.  We selected OLR after carefully evaluating its assumptions. We confirmed no multicollinearity among predictors (VIF < 5 and pairwise correlations r < .66), and the proportional odds assumption was supported by the parallel lines test (p > .05). This analytic approach allowed us to answer the research question regarding which factors predict more positive patient safety ratings, in a statistically appropriate and robust manner **(Line 249-256).** |
|  | 4. In line 285 your finding “Compared with supervisors and other clinical staff, midwives were significantly less likely to rate patient safety positively,” why this has happened? Would you explain the tool you used (if you can tool). | Thank you for your feedback. As noted, midwives were significantly less likely than supervisors and other clinical staff to rate patient safety positively, with an adjusted odds ratio (AOR) indicating a 79.2% lower likelihood (AOR = 0.208, 95% CI: 0.060–0.713, p = 0.013). This finding was based on the ordinal logistic regression (OLR) analysis, interpreted according to the adjusted odds ratio output. We used the HSOPSC 2.0 tool, categorising professional roles as nurses, physicians, midwives, pharmacy and laboratory technicians, supervisors, and other clinical staff. The bivariate OLR analysis identified a significant association (p < 0.05), and this variable was subsequently included in the multivariate OLR model, as presented in **Table 4.** |
|  | 5. Do you think that “lack of a patient safety incident reporting system,” is the main challenge is this how do you measure? | Thank you for your feedback. The lack of a patient safety incident reporting system was identified as a major challenge to ensuring patient safety in Ethiopian public hospitals. This finding emerged from the open-ended comment section of the HSOPSC 2.0. We thematically categorised the responses, with 17 participants (28%) specifically mentioning the absence of an incident reporting system **(Line 360).** |
|  | 6. I do have question on your exclusion criteria do you think that the absent of study participant during study period exclusion criteria? | Thank you for your valuable feedback. We agree that, given our use of consecutive sampling, which included all healthcare professionals who were available and consented to participate during the data collection period—the exclusion of individuals on extended leave (e.g., annual, sick, or maternity leave) occurred by default. This is due to the paper-based nature of the survey, which required the physical presence of participants in healthcare facilities. Accordingly, we have removed the corresponding statement from the manuscript stating, “Healthcare professionals on extended leave (e.g., annual, sick, or maternity leave) during the data collection period were excluded”. |
|  | 7. Do you think is it possible two ethical clearance for one research work? | Thank you for your feedback. As the research team included members from two distinct institutions across two countries (Ethiopia and Australia), and the first author was enrolled at an Australian university while conducting the study in Ethiopia, it was necessary to obtain ethical approval from both institutions. There was no conflict between the two ethics approvals, as both adhered to international human research ethics guidelines, ensuring alignment and compliance with ethical standards. |
|  | **General comment for researchers**: I have read your finding, and which is very good work, but I recommended your study population was patients because the key stakeholder due to understand or answer your research title the actors were better to take patients. | Thank you for your recommendations. Given the HSoPSC 2.0 tool is designed only for healthcare providers, it was not possible to include the patients as participants in this study. We also recognised the dual importance of healthcare providers, patients (and families) in maintaining patient safety. The recommendation to incorporate the patient perspective in assessing patient safety culture in healthcare facilities for future research is valid. For future work we will consider using standard tool such as Patient Measure of Safety (PMOS) tool to examine the patient perspectives on patient safety. We have also undertaken qualitative interviews with patients and families to explore patient safety concepts and specifically incident reporting in Ethiopian hospitals; this work will be published separately. In this study we have incorporated the recommendations to involve patients and families at policy and practice level to enhance safety and quality in healthcare **(Line 443 to 445 & Line 587).** |
| **Reviewer #4** | Dear sir, The study examined ‘Patient safety culture in resource-limited settings: insights from healthcare professionals through a multi-centre survey’. This manuscript is well-written and sound, offering a significant understanding of patient safety culture in Ethiopian public hospitals. The study effectively identifies gaps in patient safety culture and provides actionable recommendations for improving it. However, several areas could be enhanced. Overall, with minor improvements, this manuscript has the potential to contribute significantly to the field of patient safety, particularly in resource-limited settings. | We sincerely appreciate your insightful feedback. |
|  | **Introduction** The transition from HSoPSC 1.0 to 2.0 is mentioned, but the discussion on how HSoPSC 2.0 improves explicitly upon the previous version could be expanded. More concrete examples of its improvements, such as added dimensions or clarified questions, would enhance the reader’s understanding. | We have added a statement outlining the improvements made in updating the HSOPSC 1.0 to HSOPSC 2.0 to enhance clarity for the reader. These improvements included wording clarity, a reduction in the number of negatively worded items, a decrease in the number of items from 42 to 32, a reduction in dimensions from 12 to 10, and the incorporation of contemporary perspectives on patient safety **(Line 89 to 96).** |
|  | Again, while the introduction mentions Ethiopia’s under-researched state, a deeper exploration of the Ethiopian healthcare system’s specific challenges could enrich the introduction. For instance, referencing infrastructure deficits, regional disparities, and the socio-political climate in Ethiopia might provide more context for the gap in research. | Thank you for your feedback. We agree that including the challenges facing the Ethiopian healthcare system enhances clarity for readers. Accordingly, we have added a statement highlighting “Systemic challenges such as inadequate infrastructure, qualified workforce shortages, weak governance, limited training opportunities, and disparities in awareness further undermine consistent safety standards and contribute to avoidable harm. Ethiopia's healthcare system, in particular, faces significant challenges including limited funding, underdeveloped infrastructure, persistent health inequities, pronounced regional disparities, and recurring socio-political instabilities, placing considerable pressure on delivering equitable, safe, and quality healthcare” **(Lines 104–111).** |
|  | **Methods and Materials** 1. The use of “consecutive sampling” is mentioned, but the rationale behind this choice could be better explained. For example, it could clarify why this technique minimizes selection bias and is suited for the context of Ethiopian public hospitals. | Thank you for your valuable and considered comments. We have clarified the rationale for using consecutive sampling in the revised manuscript. “This approach was chosen for its practicality and suitability to the Ethiopian healthcare context, allowing for efficient recruitment without the need for a complete sampling frame (e.g., a full list of healthcare professionals in each clinical unit) unlike other probability sampling such as simple random sampling. Furthermore, consecutive sampling helps to minimise selection bias by avoiding subjective selection and including all accessible participants. It ensured that the sample reflected the routine flow of participants at the study sites and enhanced representativeness within the constraints of time and available resources **(Lines 160 and 169).** |
|  | 2. Was the tool pre-tested? While the HSoPSC 2.0 tool was adapted to local contexts, it would be helpful to discuss the specific modifications made to the tool (e.g., changes to the wording of questions or scale adaptation) to ensure its appropriateness for Ethiopian healthcare settings. | We are grateful for your detailed and constructive observations. As noted between **lines 194 and 202,** the Hospital Survey on Patient Safety Culture (HSOPS) 2.0 has demonstrated optimal validity and varying level of reliability across diverse contexts including in Italy, Brazil, China, India, Indonesia, Malysia, Norway, and South Korea. While in Chile and Australia, the tool underwent model modifications based on psychometric evaluations.  In this study, the tool was adopted with minor modifications to the background questions, based on a reviews and input from senior clinicians familiar with the local healthcare systems and context. In addition, we followed the guidance provided by the tool developer, the Agency for Healthcare Research and Quality (AHRQ), which permits adaptation of background questions to suit specific settings. Similarly, the reviewer noted that the core items were clearly worded and required no changes, as outlined between **lines 203 to 214.** We also acknowledge that comprehensive cultural adaptation and psychometric evaluation of the tool within the Ethiopian healthcare context are essential, and we have recommended this as an area for future research **(Line 623-624).** |
|  | **Results** 1. The manuscript mentions “small to medium effect sizes (η²)” but does not provide specific values, which would help the reader interpret the magnitude of differences observed. | Thank you for your feedback. The *eta squared (η²)* value for patient safety culture dimension included in ANOVA are mentioned under Table 3 with 95% Confidence Interval. We have included the magnitude of differences observed with cut of point as with small (0.01 ≤ η² < 0.06) to medium (0.06 ≤ η² < 0.14) effect sizes (η²)**(Line 318).** |
|  | 2. The manuscript should emphasize the practical significance of findings and statistical significance. For example, it could describe the implications of midwives’ lower ratings of patient safety culture beyond the statistical significance. | Thank you for your constructive feedback. We have explored and outlined the practical implication of the significant disparities in positive patient safety ratings by midwives, which was 79.2% less than supervisors and other clinical staff. Moreover, practical recommendations which can improve the perceptual gaps among healthcare professionals’ group have been incorporated. These including continuous education and simulation-based training such as regular patient safety workshops and e-learnings; promoting intra- and interprofessional collaboration through joint team huddles and shared case review meetings; fostering open communication through regular debriefings; adopting a blame-free and non-punitive approach by implementing anonymous incident reporting systems; and allocating adequate funding for education, training, and staffing **(Line 504 to 518).** |
|  | 3. Why content analysis? Which questions demanded open-ended responses? | Thank you for raising this question. Participants provided feedback in the final section of the HSoPSC 2.0 tool, which allowed participants to enter open-ended free text responses, and this was not associated with any specific question. We used content analysis as it is particularly well-suited for analysing brief, textual data where responses may not be rich in narrative detail but still offer meaningful insights on challenges in ensuring patient safety, preserving the authentic voice of participants (<https://www.sciencedirect.com/science/article/pii/S0002945923016054>). Thus, its use in this context strengthens the overall interpretation of our findings and supports a more comprehensive understanding of participants’ views on patient safety culture. |
|  | 4. Which bivariate analysis was done? Correlation? Any results for that? | Thank you for this question. We have conducted bivariate ordinal logistic regression analysis. Variable significantly associated at P-value <0.05 with outcome variable were included in multivariate ordinal logistic regression as outline under **Line 323 to 332**. For concise presentations we have only included the multivariate analysis output in **Table 4**. |
|  | **Discussion**  1. While the study draws comparisons with other settings, a more profound synthesis of the results with existing theoretical frameworks on patient safety culture would strengthen the discussion. Connecting the results to safety culture models could add depth to the interpretation. | Thank you for this suggestion. We had already incorporated the importance of utilising evidence-based frameworks such as SBAR (situation, background, assessment, and recommendation) and TeamSTEPPS (Team Strategies and Tools to Enhance Performance and Patient Safety) in providing training for staff and interventions aimed to enhance communication and teamwork, which in turn enhance patient safety **(Line 435-439).**  Furthermore, in the revised manuscript we have highlighted an evidence-based model, such as the High Reliability Organization (HRO) model, can strengthen just culture in healthcare when integrated into training and patient safety improvement initiatives. **(Line 465-472)** Additionally, to address system- and process-related challenges, the Donabedian model can be valuable. We have added additional information relating to the potential application of these evidence-based frameworks and models to enhance patient safety culture **(Line 509- 511).** |
|  | 2. Some limitations, such as the poor internal consistency of specific subscales, are briefly mentioned. These should be more deeply integrated into the discussion, particularly in how they affect the interpretation of the findings and the robustness of the conclusions. | Thank you for your feedback. We have included the potential effect of the suboptimal internal consistencies observed in some domains such as *Handoffs and Information Exchange* and *Reporting patient safety Events* **(Line 406-407).** |
|  | **Strengths and Limitations**  The internal consistency issues with several key subscales (e.g., Staffing and Work Pace) should be addressed more critically. A more precise explanation of how these limitations might have impacted the findings and their practical implications is needed. | Thank you for your valuable comments. We have incorporated notable methodological limitation pertains to the marginal to poor internal consistency of several subscales, as evidenced by Cronbach's alpha coefficients ranging from 0.19 (*Staffing and Work Pace)* to 0.80 (*Communication about error),* with multiple subscales falling below the conventional threshold of 0.7 or the HSoPSC 2.0 developer’s threshold of 0.6. These suboptimal reliability estimates indicate considerable measurement error within these constructs, which may attenuate the true relationships observed in both the ANOVA and ordinal logistic regression analyses. As a result, the effect sizes associated with these variables are likely underestimated. Therefore, the significance levels reported in these analyses should be interpreted with caution, as low internal consistency may have reduced statistical power to detect true effects, thereby increasing the risk of Type II errors. The details are included from **Line 554-569.** |
|  | **Conclusion and Implications**  While the conclusion outlines essential policy and practice recommendations, it could benefit from more emphasis on the long-term sustainability of these changes. For example, how might these recommendations be scaled or maintained over time, especially in resource-limited settings? | Thank you for your valuable suggestion. We have included the resource conscious and practical recommendations to scale-up and sustain the policies in enhancing patient safety culture in resource limited settings **(Line 588-602).** |
|  | The call for future research could be more specific. For instance, it would be beneficial to suggest follow-up studies focusing on the long-term impact of the interventions recommended in this study. | Thank you for your valuable and considered comments. We have incorporated the call for future research in exploring the long-term impact of the interventions recommended in this study.” Interventional studies should also evaluate the effectiveness of targeted strategies to improve patient safety culture in resource-limited settings. Further, follow-up studies are needed to assess the scalability and sustainability of implemented strategies to ensure their long-term effectiveness” **(Line 618-627).** |
| **Reviewer #5:** | •Thank you for your important study on patient safety culture in Ethiopian hospitals.  It is a lovely, well-written study, and I wish to be published. Your findings highlight critical gaps, especially in ICUs, and provide a strong foundation for improvement.  To build on this work, I recommend developing a corrective action plan (e.g., training and reporting systems) and remeasuring PSC annually to track progress.  •A national PSC survey could help benchmark hospitals across Ethiopia -which I am not sure is being done- while international comparisons (e.g., with LMICs/HICs) may identify best practices. | Thank you for your kind words and constructive feedback. We have already incorporated the recommendations regarding the need for cost-effective trainings through online or e-learning approach, local expertise and on-job sessions. Moreover, active engagement of frontline staff in the development of context sensitive patient safety solutions to enhance sense of ownership. Additionally, regular and periodic assessment of patient safety culture enhance the understanding of changes overtime and effectiveness of intervention. Benchmarking the results against the national and international standards can further assist in tracking the progress **(Line 385-422).**  Furthermore, we have included the benchmarking of the current study finding of overall patient safety rating against previous local study findings as well as against international study findings such as the Agency for Healthcare Research and Quality (AHRQ) user database, Organisation for Economic Co-operation and Development (OECD) countries, Brazil, China, Malysia, Saudi Arabia, and India. These studies also used the HSoPSC 2.0 tool, which is currently recommended for use to assess the patient safety culture. This comparison enhances the clarity and status of patient safety rating in Ethiopia against the international findings. Additional figure has been added as **Supplemental File 2** for details. |
|  | •Future qualitative studies could explore why ICU scores lag and how midwives perceive safety differently. | Thank you for your feedback. We have incorporated recommendations “ qualitative in-depth exploration of healthcare professionals’ perceptions and experiences regarding patient safety and incident reporting in the ICU is essential for understanding factors contributing to disparities in patient safety ratings” **(line 620-622).** This qualitative work has been conducted by our team with data currently under analysis. |
| **Reviewer #6:** | •General Comments  This manuscript addresses a critical and timely issue—patient safety culture in resource-limited healthcare settings. The authors have gathered empirical insights through a multi-center study in Eastern Ethiopia, utilizing the HSoPSC 2.0 survey instrument. The manuscript is well-written and organized, employing a methodological approach that combines quantitative analysis with qualitative insights. The statistical analyses conducted, including ordinal logistic regression and robust ANOVA techniques, enhance the credibility of the findings.  However, several methodological, analytical, and interpretative elements need clarification or enhancement to maximize their contribution. The revisions suggested below will strengthen transparency, reproducibility, and practical applicability. | Thank you for your valuable and considered comments. |
|  | **Survey Instrument**  •The selection of the HSoPSC 2.0 tool is appropriate and aligns well with international patient safety research practices. However, the manuscript currently provides limited information regarding its adaptation for use in Ethiopia. The authors are encouraged to give a detailed account of the adaptation process, including any cultural and linguistic adjustments made and the formal validation procedures, such as cognitive testing or pilot implementation, that were undertaken. This information is crucial given the low internal consistency noted in several subscales (Cronbach's alpha <0.6) and will significantly enhance the reproducibility and robustness of the study. | The tool was administered in English, as it is the medium of instruction in higher education institutions in Ethiopia (given the study included healthcare professionals with bachelor’s degree and above) and working language in hospitals. Moreover, the tool was undergone a review by senior clinicians before commencement of data collection. The review focused on the wording clarity and instructions. Therefore, few amendments were made on the background section of the tool to improve clarity and consider the contextual relevance. Moreover, instructions were included and modified in each section of the survey. However, the main questions or items remained unchanged in accordance with the tool developers’ recommendations**. (Line 207-214).** The tool demonstrated acceptable level of validity and reliability in various healthcare contexts including in Italy, Brazil, China, India, Indonesia, Malysia, Norway, and South Korea. While in Chile and Australia, the tool underwent model modifications based on psychometric evaluations **(Line 97-101).**  We agree that low internal consistency in several subscales, particularly Staffing and Work Pace (α = .19) and Handoffs and Information Exchange (α = .48)—poses a significant methodological limitation. In response, we have revised the limitations section to explicitly state how this measurement issue may have affected the results. Specifically, we now clarify that these low alpha values may have obscured true effects in both the ANOVA and ordinal logistic regression analyses, potentially resulting in non-significant findings where meaningful relationships may exist. This measurement error increases the risk of Type II errors and may have led to the underestimation of effect sizes. The revised discussion **(Lines 554-569)** reflects this concern in detail and recommends future scale refinement. |
|  | **Data Collection Methods**  The authors have provided a clear outline of the data collection procedures, which enhances the manuscript's transparency. However, additional details regarding the consent process, particularly how verbal consent was standardized and documented, are needed to bolster the study's validity further. | Thank for bringing this into our attention. The consent process included both written and verbal consent. Participants received the information sheet and consent form, which included the study’s purpose, procedures, and duration of the survey, voluntary participation, participant anonymity, data confidentiality, storage security, the right to withdraw at any time without consequences, potential benefits and risks, and contact information for the researcher team and office of ethics committees in case of any questions or concerns. Therefore, after reading the full information consent and returned the signed consent form, they received the questionnaire. However, some participants preferred not to sign the consent form but were willing to participate in the study and confirm their willingness through verbal consent, which was an additional approach to consent approved by the Institutional Ethics Review Boards. These were standardised as consistent approach was followed for all participants throughout the data collection process using the verbal consent tracking sheet, which included date, and unique and anonymous identifier without linking to personal identifier **(Line 277-280).** |
|  | Moreover, clarity on whether survey responses were anonymous or coded would help readers assess potential biases. | The survey was conducted anonymously, with no collection of participant name or other personal identifiers. After the return of the completed questionaries, unique identifier (ID) was given to each questionnaire before data entry was performed. This was helpful to cross-check the quality of entered data on computer software or to verify if there is any mistake happened during data entry **(Line 176-179).** |
|  | The training procedures for research assistants also require further clarification to ensure that other researchers can fully replicate the data collection methods. | The research assistants received structured training that covered the purpose of the study, ethical considerations, data collection procedures, key ethical considerations, and a detailed review of the questionnaire items to ensure consistency during data collection. The detailed training processes and contents were outlined under **Line 216-221.** |
|  | **Data Availability**  •The manuscript states that data will be available upon reasonable request. However, PLOS ONE strongly encourages or requires public sharing of underlying data to ensure transparency, reproducibility, and open science principles. Depositing de-identified datasets into a publicly accessible repository or providing clear justification if restrictions apply would significantly enhance the manuscript. | Thank you for raising this concern. We fully support and agree with the PLOS ONE data sharing and open science principles. As part of the journal’s editorial requirements, we confirm that the de-identified dataset will be shared in a publicly accessible repository upon acceptance of this manuscript. The link to access the data will be provided and published alongside the article. |
|  | **Sampling Strategy and Hospital Selection**  **•**The manuscript would greatly benefit from additional clarity regarding the participating hospitals' selection criteria. Whether hospitals were chosen for geographic diversity, operational capacity, patient volume, or convenience is unclear. Clarifying the rationale behind hospital selection will help readers better assess the generalizability and validity of the study's comparisons across clinical units and hospitals. | Thank you for your feedback. We have added a statement clarifying the purposive selection of five public hospitals in eastern Ethiopia, considering their operational capacity, service diversity, feasibility for data collection, administrative willingness, while also taking into account resource and time constraints.  New text included: ” Participating hospitals were purposively selected based on their operational capacity, diversity of services, willingness to participate, feasibility of data collection, and financial and time availability” **(Line 158-160).**  Furthermore, we acknowledge the limitations in generalisability to the national level, as the study was conducted in public hospitals located in eastern Ethiopia. However, the comprehensive methodology employed may serve as a useful framework for conducting a nationwide survey on patient safety culture **(Line 550-554).** |
|  | **Sociodemographic Characteristics**  •The authors provide clear and descriptive demographic characteristics of their sample. However, the sampling frame itself needs further explanation. Given that only 686 individuals were approached from a larger potential pool (>1,500 healthcare professionals), additional clarification is required regarding how this specific subset was determined and whether the sample was proportionally representative across roles, departments, or shifts. A more detailed account of the sampling frame will help readers understand potential selection bias and interpret findings. | Thank you for your feedback. Regarding the sample size, we used the single population proportion formula. The sample was proportionally allocated to each hospital based on the total number of healthcare professionals. We apologise for the typing error for the total number of healthcare professionals across five hospitals presented as 1,500 in manuscript. The correct number is 1,458 and we have replaced in manuscript as more than 1,400 healthcare professionals for simplicity **(Line 138).** For further clarity, we have included the corresponding proportional allocation as follows “Accordingly, from Hospital A, out of a total of 612 healthcare professionals, 288 were proportionally allocated. For Hospital B, 186 professionals were available and 88 were allocated; Hospital C had 165 professionals with 77 allocated; Hospital D had 308 with 145 allocated; and Hospital E had 187 with 88 allocated” **(Line 155-158).** |
|  | **Dimensions of Patient Safety Culture**  **•**The manuscript provides thorough and detailed reporting of composite and item-level scores, consistent with AHRQ recommendations. However, the significant variability within certain composites, particularly Staffing and Work Pace, requires deeper interpretative discussion. The authors should explicitly acknowledge the suboptimal internal reliability of multiple dimensions as a methodological limitation and discuss how it may impact the validity of the conclusions. | Thank you for your thoughtful feedback. We agree that low internal consistency in several subscales—particularly *Staffing and Work Pace* (α = .19) and *Handoffs and Information Exchange* (α = .48)—poses a significant methodological limitation. In response, we have revised the limitations section to explicitly state how this measurement issue may have affected the results. Specifically, we now clarify that these low alpha values may have obscured true effects in both the ANOVA and ordinal logistic regression analyses, potentially resulting in non-significant findings where meaningful relationships may exist. This measurement error increases the risk of Type II errors and may have led to the underestimation of effect sizes. The revised discussion **(Lines 554-569)** reflects this concern in detail and recommends future scale refinement. |
|  | **Benchmarking**  **•**A critical omission in the manuscript is benchmarking against international or regional norms derived from the AHRQ HSoPSC 2.0 database. Without these comparisons, whether the overall score (47% positive response) indicates severe deficiencies or is consistent with broader LMIC or global patterns remains unclear. Benchmarking the results against available international or regional data is crucial as it would significantly enhance the manuscript's contribution by providing essential context for interpreting findings. | Thank you for the valuable feedback. To strengthen the manuscript’s contribution to policy and practice, we have incorporated benchmarking data from multiple countries and regions. This includes the 2024 Agency for Healthcare Research and Quality (AHRQ) user database report, which reflects responses from 445 hospitals and 284,036 participants across the USA; the OECD report on comparative assessment of patient safety culture performance (2020–2023); and additional studies that utilised the Hospital Survey on Patient Safety Culture (HSOPSC 2.0) from Saudi Arabia, Malaysia, Brazil, China, and India. The comparative synthesis highlights both shared strengths and recurring gaps across key patient safety culture dimensions. A detailed synthesis has been added to the discussion section **(Lines 392–424)** to reflect these findings. For details see **Supplemental File 2.** |
|  | **Patient Safety Rating and Incident Reporting**  •The high rate of non-reporting (56%) identified in the manuscript raises essential concerns about reporting culture. The authors are encouraged to provide a more in-depth analysis exploring why underreporting is prevalent. Integrating these findings with qualitative responses and related subscales (such as Response to Error and Communication Openness) would offer valuable insight. The authors should discuss underreporting explicitly in terms of organizational or cultural factors, such as fear of blame or punitive responses, to add interpretative depth and practical implications. | We sincerely appreciate your insightful feedback. We have critically synthesised the study’s quantitative findings such as the low rates of patient safety incident reporting and low positive response scores in key dimensions like open communication, patient safety event reporting, and response to error with qualitative insights highlighting a blame- and shame-oriented culture, poor management support, and weak feedback mechanisms. Additionally, we have identified and discussed barriers to incident reporting across organisational, systemic, and staff-related domains, alongside key enablers. These have been integrated with actionable recommendations tailored to resource-limited settings, aimed at fostering a just culture and strengthening incident reporting practices **(Line 458-472).** |
|  | **Comparison Across Clinical Unit**  **•**The methodological rigor in comparing clinical units, particularly the use of Welch's ANOVA and Games-Howell post hoc tests, is commendable. The consistent finding of lower patient safety scores in ICUs is highly significant. To enhance interpretative value, the authors should expand the discussion of these findings by referencing ICU-specific factors such as workload intensity, staff-patient ratios, complexity of care, and institutional resources in Ethiopia. This deeper contextualization will strengthen practical relevance. | Thank you for your valuable feedback. We have addressed the challenges that exacerbate patient safety in ICU settings within Ethiopian hospitals, including severe resource constraints, inadequate staffing, weak enforcement of infection prevention protocols, insufficient staff training and capacity building, and high workloads. These issues highlight the need for context-specific interventions to enhance patient safety culture and improve the quality of care in ICUs. As such, we have included detailed, practical, and cost-sensitive recommendations that can help enhance patient safety in ICU settings **(Lines 477-494).** |
|  | **Predictors of Safety Ratings**  The ordinal regression analysis is clear, rigorous, and appropriate for the study's aims. However, reporting of model fit statistics (e.g., pseudo-R²) would enhance methodological transparency and interpretative confidence. | Thank you for your feedback. We have incorporated the detailed model fit, goodness of fit tests, pseudo-R-squared values (Cox and Snell, Nagelkerke, and McFadden ) and overall judgement on model fit and explanatory power **(Line 333-341).** |
|  | Additionally, the significant finding that midwives report lower safety ratings than other professional groups merit deeper exploration. The authors are encouraged to interpret this finding by discussing professional roles, workload distribution, decision-making autonomy, and resource allocation for midwives, highlighting practical intervention opportunities. | Thank you for your feedback. The lower perceptions of midwives on patient safety culture compared to other clinical staff in current study findings may indicate the overall working conditions in which midwives providing services, which often high workload and limited management support and allocation of resources. We have critically addressed these issues and included practical recommendations to enhance the perception of midwives on patient safety culture and bridge the perceptual gaps among healthcare professionals **(Line 504-518).** |
|  | **Open-Ended Responses**  Including open-ended responses enriches the manuscript with qualitative insights, reinforcing key quantitative findings such as resource scarcity and punitive safety culture. However, the manuscript would benefit from more straightforward thematic integration of these qualitative comments into the broader discussion. Explicitly connecting these comments to quantitative findings will help illustrate real-world impacts and improve the manuscript's depth and applicability. | Thank you for your feedback. The open-ended responses have been thematically categorised, and the frequency and percentage of comments are presented in the results section under the sub-topic “*Challenges in Ensuring Patient Safety*.” These findings highlight critical challenges related to resources, management and systems, awareness, and communication. We have incorporated and triangulated these qualitative findings with the quantitative results, as they corroborate and complement each other in the discussion section. For example, the quantitative data identified more than half of participants (56%) never reported patient safety incidents, while the open-ended responses revealed issues such as the lack of incident reporting systems and a blame-oriented organisational culture. Therefore, we have included both sets of findings under the discussion section to illustrate their convergence. Additionally, we have revised the discussion throughout to strengthen the triangulation of the quantitative and qualitative findings. |
|  | **Discussion and Literature Engagement**  The authors engage effectively with international literature. However, engagement with prior Ethiopian studies on patient safety using earlier versions of the HSoPSC tool is somewhat limited. The manuscript would significantly benefit from a deeper integration of previous national studies to identify progress or stagnation in patient safety culture, providing valuable local context and continuity in the discussion. | Thank you for bringing this to our attention. We have incorporated the previous study results from Ethiopia, published from 2016 to 2024 across various regions of the country. The findings indicated stagnant progress in patient safety rating. Studies also consistently used the HSoPSC 1.0 despite the recommendation to use the updated version, HSoPSC 2.0 **(Line 387-390).** |
|  | **Implications and Conclusion**  The implications section is well-developed and offers thoughtful recommendations. However, to fully explore the study's implications, authors are encouraged to prioritize recommendations based on feasibility, urgency, and cost-effectiveness to enhance practical utility. Identifying immediate, actionable interventions suitable for resource-limited settings will increase the manuscript's practical value. Additionally, the conclusion should be sharpened to avoid repetition, emphasizing key findings, unique contributions, and clear, actionable recommendations, particularly concerning ICU-specific strategies and leadership roles. | Thank you for your valuable and thoughtful comments. We have revised the recommendations throughout the discussion section to ensure they are practical, actionable, and grounded in the realities at the grassroots level. These recommendations aim to strengthen patient safety culture in resource-limited settings. Additionally, considerations for the sustainability and scalability of these recommendations have been included **(Line 588-617).** Additionally, we have revised the conclusion section as outlined between **line 632-649.** |
|  | **Reproducibility Note**  An overarching concern is the limited methodological detail, especially concerning survey adaptation, hospital selection, and data collection protocols. Expanding these sections to ensure other researchers can fully replicate the study methods is critical for scientific rigor, transparency, and broader applicability of the findings.  **Overall Recommendation**  This manuscript is valuable to the literature on patient safety culture in resource-constrained settings. With revisions focused on benchmarking, methodological transparency, thematic integration, and deeper contextual interpretation, it can substantially impact practice, policy, and research. The requested revisions are intended to support the authors in strengthening the manuscript's rigor and maximising its scholarly and practical contributions. | Your feedback is highly appreciated and has been instrumental in refining our work. We have carefully and thoroughly addressed each point raised by the reviewers. We sincerely appreciate their critical, scientifically sound, genuine, and constructive comments, which have significantly strengthened the quality of our work and its potential implications for policy, practice, and research. |
